# Clinical features and prognosis of patients with metastatic ocular and orbital melanoma: A bi‐institutional study

**DOI:** 10.1002/cam4.6273

**Published:** 2023-07-06

**Authors:** Xin Liu, Han Yue, Shiyu Jiang, Lin Kong, Yu Xu, Yong Chen, Chunmeng Wang, Yan Wang, Xiaoli Zhu, Yunyi Kong, Xiaowei Zhang, Jiang Qian, Zhiguo Luo

**Affiliations:** ^1^ Department of Head & Neck tumors and Neuroendocrine tumors Fudan University Shanghai Cancer Center Shanghai China; ^2^ Department of Oncology Shanghai Medical College, Fudan University Shanghai China; ^3^ Department of Ophthalmology Eye & ENT Hospital of Fudan University Shanghai China; ^4^ Department of Lymphoma Fudan University Shanghai Cancer Center Shanghai China; ^5^ Department of Radiation Oncology, Shanghai Proton and Heavy Ion Center Fudan University Cancer Hospital Shanghai China; ^6^ Shanghai Key Laboratory of radiation oncology Shanghai China; ^7^ Shanghai Engineering Research Center of Proton and Heavy Ion Radiation Therapy Shanghai China; ^8^ Department of Musculoskeletal Oncology Fudan University Shanghai Cancer Center Shanghai China; ^9^ Department of Radiation Oncology Fudan University Shanghai Cancer Center Shanghai China; ^10^ Department of Pathology Fudan University Shanghai Cancer Center Shanghai China; ^11^ Department of gastrointestinal medical oncology Fudan University Shanghai Cancer Center Shanghai China

**Keywords:** conjunctiva melanoma, lacrimal sac melanoma, ocular melanoma, orbital melanoma, uveal melanoma

## Abstract

**Purpose:**

Metastatic ocular and orbital melanomas are extremely rare. The clinical characteristics and standard treatments for these patients are not fully established.

**Materials and Methods:**

We retrospectively analyzed patients with metastatic ocular and orbital melanoma from Fudan University Shanghai Cancer Center and Eye & ENT Hospital of Fudan University between January 2012 and May 2022.

**Results:**

Overall, 51 patients with metastatic ocular and orbital melanoma were included. The most common primary sites were uvea (73%), followed by conjunctiva (22%), lacrimal sac (4%), and orbit (2%). Patients with uveal melanoma (UM) had a significantly younger age (48 vs. 68 years, *p* < 0.001), higher incidence of liver metastases (89% vs. 9%, *p*<0.001), a lower incidence of lymph nodes metastases (16% vs. 46%, *p* = 0.043) and a lower incidence of *BRAF* mutation (0% vs. 55%, *p*<0.001) compared with patients with conjunctival melanoma (CM). The overall response rate of the first‐line treatment was 18%. Three of the four patients with *BRAF*‐mutated CM responded to dabrafenib and trametinib treatment. The median progression‐free survival (PFS) and overall survival (OS) of first‐line treatment were 5.1 and 11.9 months, respectively. Among patients with liver metastases, liver‐directed treatment was correlated with better patient PFS (*p* < 0.001) and OS (*p* < 0.001) after adjusting for number of metastatic sites and primary sites.

**Conclusion:**

CM and UM have different characteristics. Patient with CM had a high incidence of *BRAF* mutation, and the treatment of BRAF and MEK inhibitors conferred clinical benefit. Liver directed therapies had a potential benefit in disease control in patients with liver metastases.

## 1 INTRODUCTION

Ocular and orbital melanomas are the second most common type of melanoma following cutaneous in western countries, which are distinct from cutaneous melanoma, with different molecular drivers, metastatic patterns and a different tumor‐immune microenvironment.^1^ Ocular melanomas include uveal melanoma (UM) and conjunctival melanoma (CM). UM accounts for more than three‐quarters of ocular melanoma, involving the choroid, iris, and ciliary body. Primary orbital melanomas are less than 1% of orbital neoplasms.^2^

For patients with localized ocular and orbital melanomas, complete surgical resection or radiation is the most important treatment. About half of the patients with UM would recur or metastasize after initial therapy. Liver is the most common involved site of metastasis.^3^ For patients with metastatic ocular and orbital melanomas, the systemic therapy showed limited efficacy without standard of care. Chemotherapy and immunotherapy exhibited poor efficacy.^4^ Tebentafusp, a T‐cell‐redirecting bispecific fusion protein, was recently approved by FDA for the treatment of previously untreated HLA‐A*02:01‐positive patients with metastatic UM.^5^

Ocular and orbital melanomas are even rarer in Asia, especially the metastatic patients.^6^ Acral and mucosal melanoma comprise about 70% of all patients with melanoma in China. Most published trials and data about ocular melanomas have arisen from western countries. Our previous study and the report from Beijing Tongren Hospital showed the tumor biology and clinical outcomes of UM might differ across ethnic groups: Asian patients have a younger onset age and tend to have a better prognosis compared to the Caucasian population.^7^, ^8^ Due to the low incidence of the disease in China, prospective studies are challenging. The clinical and pathological features of metastatic ocular and orbital melanomas are not clear yet, as well as the optimal systemic treatment options. Therefore, we conducted a bi‐institutional retrospective analysis to explore the treatment and outcome of metastatic ocular and orbital melanoma patients.

## 2 MATERIALS AND METHODS

Medical records of consecutively treated patients with metastatic ocular and orbital melanomas at Fudan University Shanghai Cancer Center and Eye & ENT Hospital of Fudan University were reviewed between January 2012 and June 2022. Patients ≥18 years old with follow‐up data available (from January 2012 to June 2022) were included. Patient characteristics, disease feature, and treatment information were recorded by reviewing the medical records. These characteristics included age, gender, *B‐RAF/N‐RAS/C‐KIT* mutational status, primary site, stage at diagnosis, sites of metastases, Eastern Cooperative Oncology Group (ECOG) performance status (PS) score, lactate dehydrogenase (LDH) level at time of first metastases, treatment, and outcomes.

Tumor response was evaluated according to Response evaluation criteria in solid tumors (RECIST), version 1.1. Progression‐free survival (PFS) was defined as the start date of first‐line therapy to date of disease progression or death from any cause, whichever was earlier. Overall survival (OS) was defined as the start date of first‐line therapy or the diagnosis of metastatic disease (for patients who did not receive any treatment) to the patient's death by any cause. Patients without any of these events by the date of latest follow‐up or study end date were censored.

Descriptive statistics were presented as frequencies with percentages for categorical variables, and median with range for continuous variables. Baseline characteristics were compared using chi‐squared testing or Fisher's exact testing for categorical variables and nonparametric rank‐sum testing for numerical variables. Neutrophil‐to‐lymphocyte ratio (NLR) was defined as absolute neutrophil count (ANC) divided by absolute lymphocyte count (ALC) and lymphocyte‐to‐monocyte ratio (LMR) was ALC divided by monocyte at baseline. Platelet‐to‐lymphocyte ratio (PLR) was calculated as platelet divided by ALC. The optimal cutoff values of baseline NLR, PLR, and LMR were determined using R package “survminer”. The Kaplan–Meier method was used to estimate survival endpoints and compared using the log‐rank test for univariate analysis. We assessed patient clinical variables using univariate and multivariate Cox proportional hazards modeling, and determined hazard ratios (HRs) and the corresponding two‐sided 95% confidence intervals (CI) for both PFS and OS. Variables with *p* < 0.1 in the univariate analysis were included in the step‐wise and final multivariate models. SPSS 27 (SPSS Inc.) and software R, version 3.3.3 (http://www.R‐project.org) were used for data analysis.

## 3 RESULTS

### 3.1 Baseline characteristics

Between January 2012 and June 2022, fifty‐one consecutive metastatic ocular and orbital melanoma patients including 37 (73%) UM, 11 (22%) CM, 2 (4%) lacrimal sac melanoma and 1 (2%) orbital melanoma were enrolled. Table 1 presented the baseline characteristics of all 51 patients. The median age was 55 years old (range 27–85) and 22 (43%) patients were male. Patients with UM had a significantly younger age at the diagnosis of metastasis (median 48 vs. 68 years old, *p*<0.001) compared with patients with CM. The first presenting symptoms recorded were diminution of vision and vision loss (38/51, 75%), followed by eyelid mass (10/51, 20%), exophthalmus (4/51, 8%), and epiphora (3/51, 6%). At the time of initial diagnosis, 4 (8%) patients had metastatic disease, and 47 (92%) patients with localized disease received radical surgery or radiation. Thirty‐two (63%) patients had *BRAF/NRAS/CKIT* mutation status available. *BRAF*, *NRAS*, and *CKIT* were mutated in 6 (12%) and 2 (4%) and 1 (3.1%) patient, respectively.

**TABLE 1:** Baseline characteristics of 51 patients with metastatic ocular and orbital melanomas.

Characteristic	Total (*N* = 51)	UM (*N* = 37)	CM (*N* = 11)	*p* value
Primary site, *n* (%)				
Uvea	37 (73)	–	–	
Conjunctiva	11 (22)	–	–	
Lacrimal sac	2 (4)	–	–	
Orbit	1 (2)	–	–	
Median age‐year (range)	55 (27–85)	48 (27–71)	68 (47–85)	**<0.001**
Sex, *n* (%)				0.270
Male	22 (43)	17 (46)	3 (27)	
Female	29 (57)	20 (54)	8 (73)	
*B‐RAF* status, *n* (%)				**<0.001**
Mutation	6 (12)	0 (0)	6 (55)	
No mutation	26 (51)	21 (57)	3 (27)	
Unknown	19 (37)	16 (43)	2 (18)	
Involved eye				0.804
Right	30 (59)	22 (60)	7 (64)	
Left	21 (41)	15 (41)	4 (36)	
ECOG PS, *n* (%)				0.255
≤1	47 (92)	33 (89)	11 (100)	
>1	4 (8)	4 (11)	0 (0)	
LDH, *n* (%)				0.421
≤ULN	18 (35)	12 (32)	5 (46)	
1–2 × UNL	8 (16)	5 (14)	3 (27)	
>2 × ULN	6 (12)	4 (11)	1 (9)	
Unknown	19 (37)	16 (43)	2 (18)	
Metastatic sites				
Liver	36 (71)	33 (89)	1 (9)	**<0.001**
Bone	15 (29)	9 (24)	4 (36)	0.430
Lung	15 (29)	10 (27)	5 (46)	0.247
Lymph nodes	13 (25)	6 (16)	5 (46)	**0.043**
≥ 3 metastatic sites	14 (27)	9 (24)	4 (36)	0.430

Bold values are statistically significant.

Abbreviations: CM, conjunctival melanoma; ECOG, Eastern Cooperative Oncology Group; LDH, lactate dehydrogenase; PS, performance score; ULN, upper limit of normal; UM, uveal melanoma.

Among 37 patients with UM, no patient had *BRAF/NRAS/CKIT* mutation. While 6 (55%) of 11 patients with CM had *BRAF* (V600E [*n* = 5], V600G [*n* = 1] mutation), 2 (18%) had *NRAS p*.Q61K mutation. One patient with *BRAF* V600E mutation had concurrent *CKIT* D579N mutation. Among two patients with lacrimal sac melanoma, next generation sequencing (NGS) was performed in one patient and revealed *NF1* mutation (Table S1).

### 3.2 Initial treatment and relapse pattern

Of the 51 patients, 21 (41.2%) patients had histological re‐confirmation for primary diagnosis. The time from radical treatment to metastatic disease ranged from 0.4 to 36.7 years, at a median of 3.9 years. Most (*n* = 45, 88%) patients were histopathologically confirmed as metastatic melanoma. The most common metastatic sites were liver (*n* = 36, 71%), bone (*n* = 15, 29%), lung (*n* = 15, 29%), and lymph nodes (*n* = 13, 25%). Only 9 of patients had ocular magnetic resonance (MR) imaging performed based on the disease involvement.

Among 37 patients with UM, most (*n* = 34, 92%) patients with localized disease received radical therapy, including 28 patients receiving enucleation and 6 receiving radiation (Iodine 125 [*n* = 4], proton radiation therapy [*n* = 1], and gamma knife radiation [*n* = 1]). None but one patient received adjuvant high‐dose interferon. All of the 11 patients with CM received radical surgery. Five patients received adjuvant treatment, including programmed cell death protein 1 (PD‐1) inhibitors (*n* = 2), high‐dose interferon (*n* = 1), radiation and PD‐1 inhibitor plus temozolomide (TMZ) treatment (*n* = 1), vemurafenib (*n* = 1). Patients with UM had higher incidence of liver metastases (89% vs. 9%, *p* < 0.001), a lower incidence of lymph nodes metastases (16% vs. 46%, *p* = 0.043) compared with patients with CM (Table 1).

### 3.3 Front‐line treatments and response

The treatment and responses of the 51 patients were presented in Table 2. The patients received a variety of first‐line treatment regimens, including chemotherapy with or without antiangiogenic therapy (*n* = 21, 41%), PD‐1 inhibitor with or without antiangiogenic therapy (*n* = 14, 27%), PD‐1 inhibitor combined with antiangiogenic therapy and chemotherapy (*n* = 7, 14%), and dabrafenib plus trametinib (*n* = 3, 6%). Three (6%) patients received liver directed therapies alone. Three (6%) patients did not receive any treatment because of clinical deterioration or refusal to treatment. Among 36 patients with liver metastases, 17 (47%) patients received liver directed therapies during first‐line treatment, including transcatheter arterial chemoembolization (TACE) (*n* = 6), radiofrequency ablation (*n* = 5), TACE and radiofrequency ablation (*n* = 4), and liver radiation (*n* = 2). The overall response rate (ORR) was 18% (9/51), with all the 9 patients achieving partial response (PR). The disease control was achieved in 33 patients (65%).

**TABLE 2:** First‐line therapies and responses to treatment.

Characteristic	Total (*N* = 51)	UM (*N* = 37)	CM (*N* = 11)
First‐line therapy, *n* (%)			
Chemotherapy ± antiangiogenesis agents	21 (41)	20 (54)	1 (9)
PD‐1 inhibitors ± antiangiogenesis agents	14 (27)	9 (24)	3 (27)
PD‐1 inhibitors plus antiangiogenesis agents plus chemotherapy	7 (14)	4 (11)	2 (18)
BRAF/MEK inhibitor combination	3 (6)	0	3 (27)
Liver directed therapies only	3 (6)	3 (8)	0
No	3 (6)	1 (3)	2 (18)
Best overall response, *n* (%)			
Complete response	0	0	0
Partial response	9 (18)	5 (14)	3 (27)
Stable disease	24 (47)	18 (49)	4 (36)
Progressive disease, *n* (%)	14 (27)	13 (35)	1 (9)
No treatment	3 (6)	1 (3)	2 (18)
Not available	1 (2)	0	1 (9)
Objective response, *n* (%)	9 (18)	5 (14)	3 (27)
Disease control	33 (65)	23 (62)	7 (64)

Abbreviations: CM, conjunctival melanoma; UM, uveal melanoma.

**FIGURE 1:**
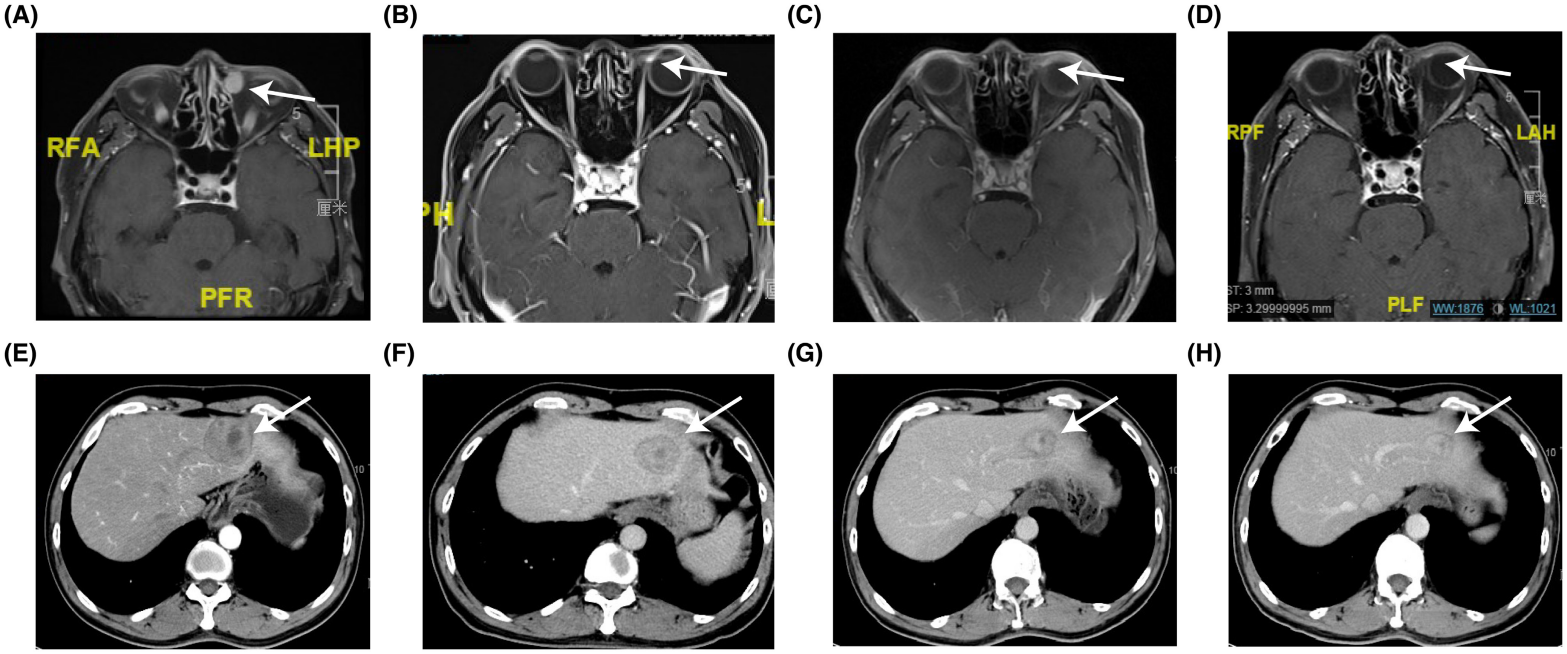
One patient with metastatic lacrimal sac melanoma who achieved partial response after pembrolizumab plus Rh‐endostatin therapy. MRI images of lacrimal sac melanoma before treatment (A), 3 months (B), 5 months (C) and 14 months (D) after treatment. CT images of liver metastases at baseline (E), 3 months (F), 5 months (G) and 7 months (H) after treatment.

Notably, one patient with lacrimal sac melanoma was treated with PD‐1 inhibitor plus Rh‐endostatin. Hepatic stereotactic body radiation therapy (SBRT) of 40Gy/5Fx was given concurrently during the systemic therapy. This patient received a PR (Figure 1) and still in response under PD‐1 inhibitor plus Rh‐endostatin treatment by the time of analysis, with a duration of disease control over 19.3 months. Nevertheless, another patient with lacrimal sac melanoma achieved SD with PD‐1 inhibitor combined with Rh‐endostatin and chemotherapy, and the PFS was 3.9 months. The only one patient with orbital melanoma in this cohort was treated with first‐line PD‐1 inhibitor plus Rh‐endostatin. He received a best response of SD and the PFS was 3.9 months. The disease continued progressed with the second‐line treatment of PD‐1 inhibitor plus nab‐paclitaxel and carboplatin.

### 3.4 Survival analysis and exploration of prognostic indicators

Among eight patients receiving second‐line treatment, PR was observed in only one patient who received dabrafenib plus trametinib. Four patients had stable disease (SD) following nab‐paclitaxel combined with carboplatin and bevacizumab (*n* = 3) and anti‐PD‐1 treatment (*n* = 1).

**FIGURE 2:**
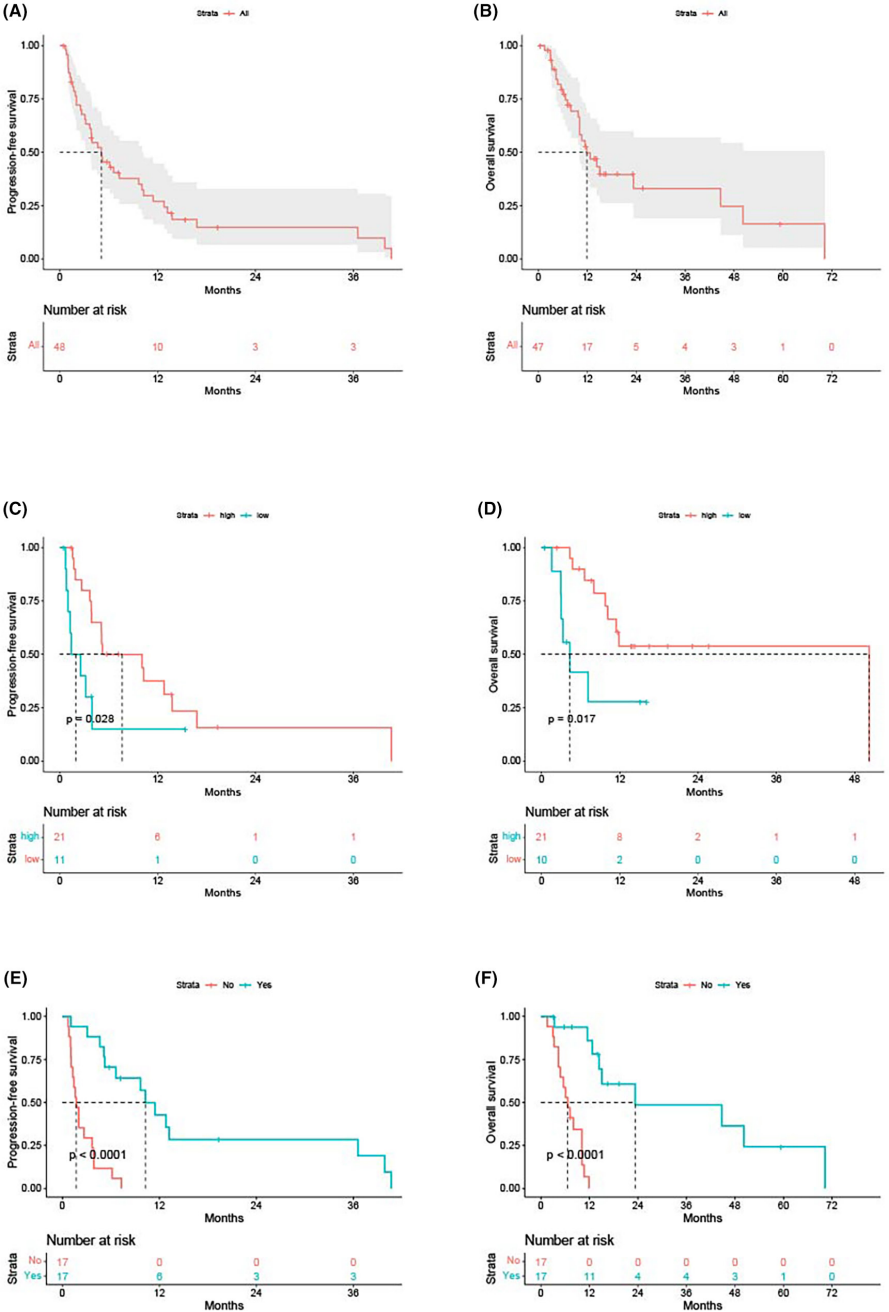
The progression‐free survival (PFS) (A) and overall survival (OS) (B) of the entire cohort. Higher LMR was associated with better PFS (C) and OS (D). Among patients with liver metastases, receipt of liver directed therapies improved patient PFS (E) and OS (F).

Of June 2022, the median follow‐up time was 16.1 months (95% CI: 13.3–18.9 months). The median PFS of first‐line treatment was 5.1 months (95% CI: 3.7–10.3 months; Figure 2A). The median OS was 11.9 months (95%CI: 10.1‐NA months; Figure 2B). In patients who had UM and CM, the median PFS were 5.1 months (95%CI: 3.0–7.2 months) and 10.1 months (95%CI: 0–20.6 months) (*p* = 0.516), the median OS were 11.5 months (95%CI: 9.3–13.6 months) and unreached (*p* = 0.142), respectively. The cutoff of NLR, PLR, and LMR were 2.1, 173.08 and 3.4, respectively. Higher LMR was associated with better PFS (*p* = 0.028, Figure 2C) and OS (*p* = 0.017, Figure 2D). No significant difference was found in PFS or OS with patient baseline NLR or PLR.

Three or more metastatic sites (HR = 4.73, 95%CI = 1.49–14.97, *p* = 0.008), higher LMR (HR = 0.30, 95%CI = 0.11–0.81, *p* = 0.017), conjunctiva/lacrimal sac/orbit as primary sites (HR = 0.30, 95%CI = 0.10–0.90, *p* = 0.032) and female patients (HR = 3.46, 95%CI = 1.39–8.62, *p* = 0.008) were independently correlated with patient PFS. Similarly, three or more metastatic sites (HR = 6.22, 95%CI = 2.28–16.78, *p* < 0.001) and conjunctiva/lacrimal sac/orbit as primary sites (HR = 0.16, 95%CI = 0.03–0.75, *p* = 0.020) were independently associated with patient OS.

In patients with liver metastases, the median PFS were 3.9 months (95%CI: 1.8–6.0 months), the median OS was 10.7 months (95%CI: 7.3–14.1 months). Among patients with liver metastases, receipt of liver directed therapies improved patient PFS (1.7 vs. 10.3 months, *p*<0.001; Figure 2E) and OS (6.6 vs. 23.3 months, *p*<0.001; Figure 2F). After adjusting for number of metastatic sites and primary sites, liver‐directed treatment was correlated better patient PFS (HR = 0.34, 95%CI = 0.05–0.29, *p* < 0.001) and OS (HR = 0.06, 95%CI = 0.01–0.28, *p* < 0.001).

## 4 DISCUSSION

The biological behaviors and treatment for patients with metastatic ocular and orbital melanomas are largely unknown. Actually, data are even rare involving Chinese patients. This present study reports the characteristics, treatment, and outcomes of 51 patients with metastatic ocular and orbital melanomas, providing real‐world data of the treatment and response of this population.

In our study, the median age was 55 years old, with a similar proportion of female and male. The most common primary lesions were uvea (73%), followed by conjunctiva (22%). The UM patients were at a similar proportion with western countries, which is higher than reported in other Asian studies (uvea: 61.3%–65.9%).^6^, ^9^ Similar to previous reports of UM in China,^7^ the mean age of patients with UM in this cohort was 48 years old, younger than that of Caucasians.^10^ Moreover, it has been revealed that the incidence of UM is higher in Whites than in Asians and Asian patients have a higher mean basal diameter compared to whites.^11^, ^12^ These difference may indicated the racial difference in UM, thus the epidemiology, the genetic makeup of tumors and prognostication of Asian patients need further study.

We found that UM and CM had distinct clinical and genomic characteristics. Patients with UM had a higher incidence of liver metastases (89% vs. 9%, *p* < 0.001) compared with patients with CM. The mechanism of liver‐predominant metastases in UM is not fully understood. Some evidence showed that cMET expression by UM cells plays a part in their affinity for the liver. It's reported that liver metastases through hematogenous spread represents up to 90% of cases of disseminated disease and is often the first and only site of metastases. On the contrary, liver metastases is not common in CM. CM are often preceded by regional lymph node metastasis. Lung, lymph nodes, and bone were the most common sites of metastases in patients with CM in our series. Lodde et al. also found the most involved sites of distant metastases at start of first systemic treatment of CM were lung and lymph nodes.^13^ Studies had showed that most patients that develop metastatic disease are diagnosed with metastasis within 5 years after initial diagnosis. However, patients are at risk of developing metastases also 20 years after the initial diagnosis. In our study, the metastatic disease was diagnosed at a median of 3.9 years after initial diagnosis. One patient developed liver metastases as long as 36.7 years after the initial diagnosis of UM. Less than 3% of patients present with metastatic disease at initial diagnosis, which is consistent with our results (4%).^14^

Patients with CM had a higher incidence of *BRAF* mutation (55% vs. 0%, *p* < 0.001) compared with patients with UM. Tan et al. also reported a different *BRAF* mutant status between UM (8.3%) and CM patients (57.1%) (*p* = 0.038) in a multi‐ethnic Asian cohort.^6^ Lodde et al. launched a multi‐center retrospective cohort study with 34 patients with metastatic CM and identified frequent mutations of *BRAF* (46.7%, 7/15) and *NRAS* (26.7%, 4/15). In our previous retrospective analysis, the most commonly observed mutations in mucosal melanoma were *NRAS* (23.1%), *BRAF* (7.7%), and *CKIT* mutations (5.1%).^15^ These evidence supports the role of UV irradiation in CM and the genetic similarity with cutaneous melanoma. On the other hand, UM has unique genetic characteristics by the presence of GNAQ/11 mutations and activation of the downstream pathways.^16^, ^17^

There is no standard first‐line treatment for patients with metastatic ocular and orbital melanomas, who are excluded from most melanoma clinical trials. Chemotherapy, immunotherapy or targeted therapy had failed to show convincing efficacy in patients with metastatic UM, with an ORR of 0%–10% and a median OS of less than 1 year.^18^, ^19^, ^20^ Combination of cisplatin, vinblastine, and dacarbazine as first line chemotherapy for liver metastatic UM showed an ORR of 20%, a median PFS of 5.5 months and a median OS of 13 months.^21^ A phase II study of nivolumab with ipilimumab in patients with metastatic UM resulted an ORR of 18%, a median PFS of 5.5 months and a median OS of 19.1 months.^22^ Other targeted therapy such as MEK and PI3K inhibitors had showed disappointing activity.^23^, ^24^ The patients in our cohort received a variety of treatment regimens, containing chemotherapy, PD‐1 inhibitor, anti‐angiogenesis agents, targeted therapies, and liver‐directed treatment. The ORR of first‐line treatment was 18% (9/51), with more responses observed in CM than UM (27% vs. 14%). Rh‐endostatin has been reported to efficiently block angiogenesis and suppress tumor growth. Our previous study showed Rh‐endostatin combined with chemotherapy in patients with advanced mucosal melanoma resulted a RR of 30.0%.^15^ Despite the efficacy is unsatisfactory in metastatic ocular and orbital melanomas in our study, the combination of PD‐1 inhibitor and Rh‐endostatin showed favorable response (ORR: 33.3%). The two responders are under the treatment of PD‐1 inhibitor plus Rh‐endostatin with a duration of response more than 1 year. The combination of Rh‐endostatin and PD1 inhibitor warrants further research in this population.

Recently reported data of tebentafusp in de novo patients with metastatic UM showed significantly longer OS and PFS compared with the control group receiving the investigator's choice of therapy with single‐agent pembrolizumab, ipilimumab, or dacarbazine,^5^ with a median PFS of 3.3 and 2.9 months (*p* = 0.01) and a median OS of 21.7 and 16.0 months (*p* < 0.001), respectively. The overall benefit of tebentafusp monotherapy seems limited and the efficacy is unclear in Asian patients.

Neutrophils can reflect the state of host inflammation and lymphocytes related to the immune response against cancer. Recently, some synthetic parameters like NLR and LMR have also emerged as biomarkers with prognostic value.^25^, ^26^ We have recently investigated the prognostic value of inflammatory indexes in patients with advanced or recurrent mucosal melanoma treated with continuous Rh‐endostatin infusion plus chemotherapy, and indicated high LMR correlated with favorable PFS and OS in this patient population. Additionally, another study involving patients with advanced melanoma confirmed the prognostic value of LMR.^27^ The present study included patients with metastatic ocular and orbital melanoma and indicated the pretreatment high LMR were associated with better survival. Further studies incorporating inflammatory indexes such as LMR into the prognostication are warranted to better stratify patient risk.

Melanoma patients with liver metastases had disappointing response to systemic therapy. And liver directed therapies including TACE, percutaneous hepatic perfusion (PHP), radiation, and ablation may bring clinical benefit in such patients.^28^, ^29^ In our study, the application of liver directed therapies prolonged patient PFS and OS, even after adjusting for number of metastasis and primary sites. Previously, Dewald et al. analyzed 66 patients with hepatic‐dominant UM treated with PHP, resulting an ORR of 59%, a median hepatic PFS of 12.4 months and median OS of 18.4 months. Similarly, a recent meta‐analysis demonstrated a longer median PFS and OS for patients who received liver‐directed therapies compared to systemic therapy.^30^ However, liver‐directed therapies may be selected in patients with proper liver function and it remains unknown which liver directed therapy is of priority. In our study, two patients who received liver radiation and immunotherapy showed a good tumor control. The response to immunotherapy depends on preexisting tumor infiltrate and may be improved by radiotherapy, which is able to increase tumor antigens visibility and promote priming of T cells.^31^, ^32^, ^33^, ^34^ Preliminary results have indicated promising results of thus combinations in terms of survival outcomes.^35^ However, further studies are needed to confirm such evidence in this patient population.

Larger prospective studies are also required to confirm the benefit of liver‐directed therapies in this patient population, as well as the selection of patients. As an aggressive disease, advanced, or metastatic disease is commonly observed in patients with metastatic ocular and orbital melanoma, and liver metastasis is the most common metastatic site. The present study has demonstrated receipt of liver directed therapies could improve patient survival. However, the liver directed therapies differed according to the number of sites, size of metastasis, the rest healthy liver tissue, and individual physical status. Thus, we believe multidisciplinary tumor board discussion could assist in optimizing the patient‐centered and integrated care and continuously improve the practice to deal with this challenging population. Treatment planning should be made with the joint effort of the professionals in the multidisciplinary tumor board. Evaluation of the patient PS, medical history, disease status, and evaluation of the liver metastasis are critical to decide the optimal treatment approach. The tumor biology, the genomic characteristics, the sensitivity to the previous treatment, and the evaluation of the liver metastasis should be taken into consideration.

In patients with B‐RAF mutated CM, the treatment of dabrafenib and trametinib resulted in an ORR of 75% in our study. The similarity between CM and cutaneous melanoma in terms of genetics makes us learn experience from the treatment of cutaneous melanoma. Patients with *BRAF*‐mutated metastatic CM have been reported to respond to *BRAF* inhibition.^13^, ^36^ However, Lodde et al. claimed patients with BRAF‐mutated CM achieved a DCR of 37.5% and a median PFS of 12.6 months following BRAF inhibitor with or without MEK inhibitor, which demonstrating that patients with CM can derive long‐term benefit from targeted therapy.^13^ The response rate of BRAF inhibition *BRAF*‐mutated metastatic CM in the present study is higher than previously reported, which may indicated the racial difference in response to BRAF inhibition. The efficacy of BRAF and MEK inhibitor and the genetic characteristics in B‐RAF mutated CM should be further explored in Chinese patients.

Malignant melanoma arising in the lacrimal sac and orbit are extremely rare, and a limited number of case reports in the literature can be listed on hand. Matsuo et al. reported a patient with lacrimal sac melanoma harboring *BRAF* V600E mutation.^37^ Adetunji et al. reviewed eighty‐eight cases of primary orbital melanoma, 42% of whom had orbital blue nevus and 36% had metastases. The efficacy of immunotherapy for primary orbital melanoma remained unknown.^38^ In our cohort, one patient with lacrimal sac melanoma showed good response to systemic therapy with Rh‐endostatin and PD‐1 inhibitor, and liver radiation.

There are some limitations of this study. First, this is a retrospective study and the sample size is relatively small. Second, due to the lack of standard first‐line treatment, the variety of treatment may result in bias. Third, only three patients with orbital melanoma made it difficult to compare with UM and CM. However, given the rarity of the disease and the limited data of Chinese patients, the result of data may bring insight for further investigations.

## 5 CONCLUSION

The results of this study suggested that metastatic ocular and orbital melanoma is a heterogeneous disease. Metastatic UM was associated with a poor prognosis, a high incidence of liver metastases, and a low incidence of *BRAF/NRAS/CKIT* mutation. Liver‐directed treatments may bring clinical benefit for such patients. Metastatic CM was associated with a better prognosis, a lower incidence of liver metastases and a high incidence of *BRAF* V600 mutation. B‐RAF and MEK inhibitors showed promising benefit in advanced CM patients with *BRAF* V600 mutation. The role of B‐RAF signaling pathway in CM and the mechanism of drug resistance are worthy of further study from clinical and basic aspects.

## AUTHOR CONTRIBUTIONS

**Xin Liu:** Conceptualization (lead); data curation (lead); formal analysis (lead); investigation (lead); supervision (lead); writing – original draft (lead); writing – review and editing (lead). **Yue Han:** Conceptualization (lead); data curation (lead); investigation (equal); writing – review and editing (equal). **Shiyu Jiang:** Conceptualization (equal); data curation (equal); formal analysis (equal); investigation (lead); writing – original draft (lead); writing – review and editing (equal). **Lin Kong:** Conceptualization (equal); data curation (equal); investigation (equal); writing – review and editing (equal). **Yu Xu:** Conceptualization (equal); data curation (equal); investigation (equal); writing – review and editing (equal). **Yong Chen:** Conceptualization (equal); data curation (equal); investigation (equal); writing – review and editing (equal). **Chunmeng Wang:** Conceptualization (equal); data curation (equal); investigation (equal); writing – review and editing (equal). **Yan Wang:** Conceptualization (equal); data curation (equal); investigation (equal); writing – review and editing (equal). **Xiaoli Zhu:** Conceptualization (equal); data curation (equal); investigation (equal); writing – review and editing (equal). **Yunyi Kong:** Conceptualization (equal); data curation (equal); investigation (equal); writing – review and editing (equal). **Xiaowei Zhang:** Conceptualization (lead); data curation (lead); investigation (equal); writing – review and editing (lead). **Jiang Qian:** Conceptualization (lead); data curation (lead); investigation (lead); supervision (lead); writing – review and editing (lead). **Zhiguo Luo:** Conceptualization (lead); data curation (lead); investigation (lead); supervision (lead); writing – original draft (equal); writing – review and editing (lead).

## CONFLICT OF INTEREST STATEMENT

The authors declare that they have no competing interests.

## ETHICS STATEMENT

The study was conducted in accordance with the Declaration of Helsinki and Good Clinical Practice guidelines. This study was approved by the Ethics Committee of Fudan University Shanghai Cancer Center (N0. 2208259‐10). All patients provided written informed consent to participated in this study and publication of the data.

## Supporting information


Table S1.

## Data Availability

All data of this study are available per the corresponding author's approval.
