# Supplementary material for: Clinical features and prognosis of patients with metastatic ocular and orbital melanoma: A bi‐institutional study
Source: Cancer Med. 2023 Jul 6;12(15):16163–72. doi: 10.1002/cam4.6273 (PMC10469730; doi:10.1002/cam4.6273)
Supplement: Supplementary file 1 — Table S1. [file CAM4-12-16163-s001.docx]

Table S1. The result of NGS of the patient tumor sample.

| ID | Quality control | Type | Status | Gene | Transcript_id | Mutation Type | hgvs_p | hgvs_c | Chrom | Depth | Af |
| --- | --- | --- | --- | --- | --- | --- | --- | --- | --- | --- | --- |
| 8888880008753 | Pass | short-variant | unknown | NTRK3 | NM_002530 | missense | R343W | 1027C>T | chr15:88678509 | 1362 | 51.84 |
| 8888880008753 | Pass | short-variant | unknown | NTRK3 | NM_002530 | missense | V308L | 922G>C | chr15:88678614 | 1188 | 29.38 |
| 8888880008753 | Pass | short-variant | unknown | TIPARP | NM_015508 | missense | R133L | 398G>T | chr3:156395884 | 1691 | 78.89 |
| 8888880008753 | Pass | short-variant | unknown | TSC2 | NM_000548 | missense | V1618I | 4852G>A | chr16:2136735 | 875 | 43.77 |
| 8888880008753 | Pass | short-variant | unknown | SETD2 | NM_014159 | missense | H2249R | 6746A>G | chr3:47098528 | 1413 | 54.99 |
| 8888880008753 | Pass | short-variant | known | NF1 | NM_001042492 | missense | M1981V | 5941A>G | chr17:29661984 | 863 | 7.07 |
| 8888880008753 | Pass | short-variant | unknown | LTK | NM_002344 | splice | splice site 657+2_657+6delTACGT | 657+2_657+6delTACGT | chr15:41804008 | 656 | 46.80 |
| 8888880008753 | Pass | short-variant | unknown | ARID1A | NM_006015 | missense | R1671W | 5011C>T | chr1:27102085 | 470 | 21.91 |
| 8888880008753 | Pass | short-variant | known | SF3B1 | NM_012433 | missense | R625C | 1873C>T | chr2:198267484 | 1194 | 26.05 |
